# Superimposed Wavefront Imaging of Diffraction-enhanced X-rays: sparsity-aware CT reconstruction from limited-view projections

**DOI:** 10.1007/s11548-024-03303-y

**Published:** 2024-12-26

**Authors:** Naoki Sunaguchi, Tetsuya Yuasa, Daisuke Shimao, Zhuoran Huang, Shu Ichihara, Rieko Nishimura, Akari Iwakoshi, Jong-Ki Kim, Rajiv Gupta, Masami Ando

**Affiliations:** 1 Department of Radiological and Medical Laboratory Sciences, Nagoya University Graduate School of Medicine, Nagoya, Aichi 461-8673 Japan; 2 Graduate School of Engineering and Science, Yamagata University, Yonezawa, Yamagata 992-8510 Japan; 3 Department of Radiological Sciences, International University of Health and Welfare, Otawara, Tochigi 324-8501 Japan; 4 Department of Pathology, NHO Nagoya Medical Center, Nagoya, Aichi 460-0001 Japan; 5 Biomedical Engineering and Radiology, School of Medicine, Catholic University of Daegu, Daegu, 705-034 Korea; 6 Department of Radiology, Massachusetts General Hospital and Harvard Medical School, Boston, MA 02114 USA; 7 High Energy Accelerator Research Organization, Tsukuba, Ibaraki 305-0801 Japan

**Keywords:** CT reconstruction, Total variation, Analyzer-based refraction-contrast CT, Superimposed Wavefront Imaging of Diffraction-enhanced X-rays (SWIDeX)

## Abstract

**Purpose:**

In this paper, we describe an algebraic reconstruction algorithm with a total variation regularization (ART + TV) based on the Superimposed Wavefront Imaging of Diffraction-enhanced X-rays (SWIDeX) method to effectively reduce the number of projections required for differential phase-contrast CT reconstruction.

**Methods:**

SWIDeX is a technique that uses a Laue-case Si analyzer with closely spaced scintillator to generate second derivative phase-contrast images with high contrast of a subject. When the projections obtained by this technique are reconstructed, a Laplacian phase-contrast tomographic image with higher sparsity than the original physical distribution of the subject can be obtained. In the proposed method, the Laplacian image is first obtained by applying ART + TV, which is expected to reduce the projection with higher sparsity, to the projection obtained from SWIDeX with a limited number of views. Then, by solving Poisson's equation for the Laplacian image, a tomographic image representing the refractive index distribution is obtained.

**Results:**

Simulations and actual X-ray experiments were conducted to demonstrate the effectiveness of the proposed method in projection reduction. In the simulation, image quality was maintained even when the number of projections was reduced to about 1/10 of the originally required views, and in the actual experiment, biological tissue structure was maintained even when the number of projections was reduced to about 1/30.

**Conclusion:**

SWIDeX can visualize the internal structures of biological tissues with very high contrast, and the proposed method will be useful for CT reconstruction from large projection data with a wide field of view and high spatial resolution.

## Introduction

Phase-contrast computed tomography (CT) is a technique to visualize the electron density distribution in an object by measuring the phase shift caused by the interaction between the object and X-rays. The physical quantity in an object is generally expressed in terms of the complex refractive index $n=1-\delta +i\beta$, where the real part *δ* is the X-ray phase shift proportional to the electron density and the imaginary part *β* is related to the interaction of X-ray absorption. In the hard X-ray region, the * δ*’s of biological soft tissues composed of light elements, such as H, O, C, and N, are approximately 1000 times larger than *β*. Therefore, phase-contrast CT has the potential to visualize the internal structure of biological soft tissues with high contrast. There are several imaging methods for phase-contrast CT, including inline holography [1], interferometry [2], grating-based imaging [3, 4], and analyzer-based imaging [5–8], and they are used for research aimed at clinical applications, microanatomy of various organs, analysis of various diseases, and so on [9–17].

The projection information obtained by phase-contrast CT depends on the imaging method: in the interferometry, it is the line integral of *δ* of the object, i.e., the amount of phase shift when X-rays propagate through the object, while in other methods, it is the derivative of the line integral of *δ*, where the derivative is with respect to the direction perpendicular to the beam propagation in the reconstruction plane. When reconstructing the distribution of *δ* within an object, the interferometry can be used directly with methods such as Filtered Backprojection (FBP), which is similar to absorption-contrast CT, but other methods require the projection to be converted to a line integral of *δ* by applying an integration process to the projection before reconstruction. On the other hand, it is also possible to obtain a differential phase-contrast computed tomography (d-PCT) by performing reconstruction without applying the integration process to the projection [18].

In recent years, the field of view has been expanded and the spatial resolution has been improved in phase-contrast CT using synchrotron radiation X-rays [19], and the number of pixels in CT images has similarly increased [20]. Normally, if blurring during imaging is ignored, the number of projection views required for CT reconstruction is π/2 times the number of pixels on one side of the CT image based on Nyquist-Shannon sampling theorem [21], so the trend toward a larger field of view and higher spatial resolution significantly increases the measurement time for a single sample. Since many cases are observed when analyzing biological samples, it is desirable to be able to reconstruct a CT image with high quality using a small number of projection views.

The d-PCT has a higher sparsity than the original CT image (distribution of *δ*), effectively reducing the number of projection views required for CT reconstruction. Our group applied an algebraic reconstruction technique (ART) using total variation (TV) regularization (ART + TV) to differential projections obtained from X-ray dark-field imaging (XDFI) based on the analyzer-based method and showed that TV regularization is effective in projection reduction for d-PCT [22]. We also proposed the ART + TV method using the second derivative projections, which takes advantage of the higher sparsity of the second derivative projections obtained by applying differencing to the first derivative projections of the XDFI [23]. This method successfully reduces the number of projection views while maintaining image quality, even for subjects with complex *δ* distributions. However, it has the problems of amplifying noise and degrading spatial resolution due to difference processing applied between adjacent pixels in the measurement data.

We recently proposed the Superimposed Wavefront Imaging of Diffraction-enhanced X-rays (SWIDeX) method, which can improve the spatial resolution over XDFI [24]. While XDFI measures the first derivative projections of the object using a Laue-type Si crystal Angle Analyzer (LAA) placed behind the object in the X-ray propagation path, SWIDeX directly measures the second derivative projections of the object by closely contacting the LAA and the scintillator. Therefore, ART + TV, which uses SWIDeX projections, may work more effectively than XDFI for projection reduction because it does not require the differencing process used to obtain sparsity in XDFI.

In this study, we propose the limited-view CT reconstruction using sparsity in SWIDeX and demonstrate its effectiveness using both simulated data and experimental data from human biological tissues.

## Method

### SWIDeX principle to generate a projection image with high sparsity

**Fig. 1:**
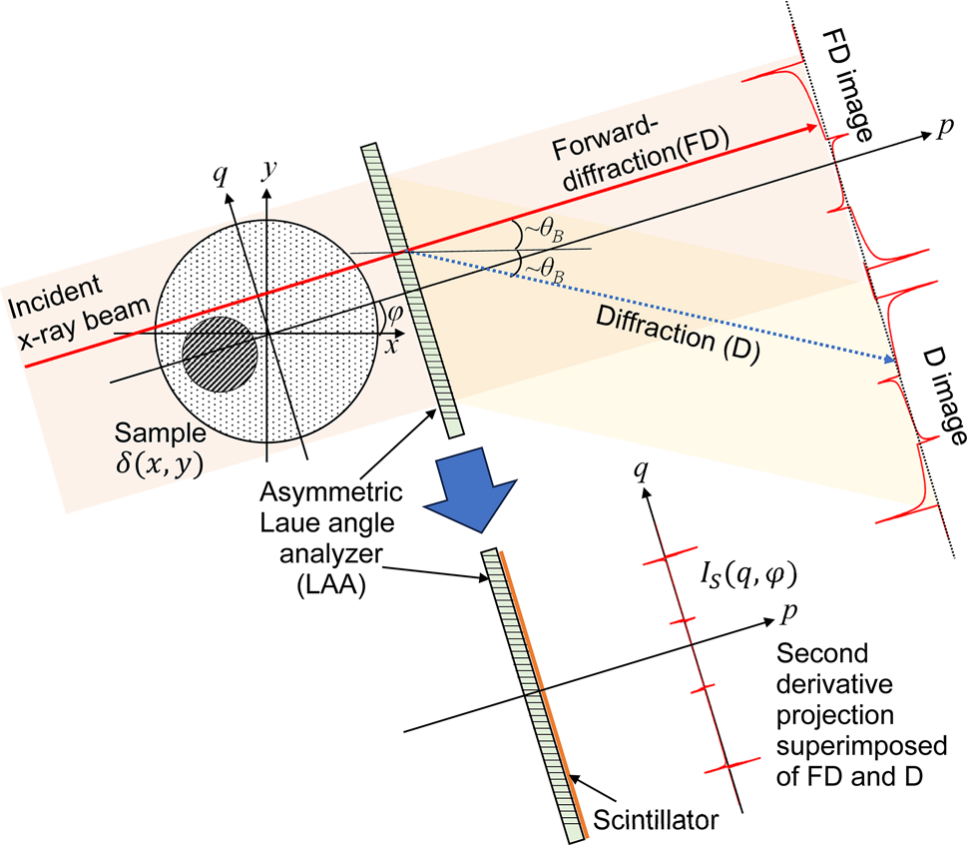
A schematic diagram of SWIDeX measurement system

Initially, we describe the principle of SWIDeX to obtain a projection representing the second-order derivative of the line integral of δ. Figure 1 is a schematic diagram of SWIDeX measurement system using LAA. In this figure, the *xy*-coordinate system is set for the subject, and the refractive index distribution $\delta \left(x,y\right)$ of the subject is placed on the *xy*-plane. The *pq*-coordinate system is obtained by rotating the *xy*-coordinate system by an angle *φ* around the origin. The incident x-ray beam propagates in the *xy*-plane parallel to the* p*-axis. The beam propagating through the subject is impinged on the LAA, which is installed parallel to the *q*-axis. When the beam enters the LAA at an angle of incidence near the Bragg angle ${\theta }_{B}$, it is divided at the back of the LAA into a Forward-Diffraction (FD), which propagates in the same direction as the direction of incidence, and a Diffraction (D), which propagates in the $2{\theta }_{B}$ direction. The FD and D X-ray intensities can be calculated theoretically from the respective rocking curves, which are functions of the beam incidence angle into the LAA. Each rocking curve has a steep gradient near the Bragg angle, which can be used to produce refraction-contrast images. When the incident X-ray energy is high and the LAA thickness is thin, the sum of the FD and D intensities is approximately equal to the incident X-ray intensity from the energy conservation law, and the FD and D produce contrasts that are inverted from each other. Each X-ray intensity can be defined using the functions ${R}_{FD}$ and ${R}_{D}$ for each rocking curve as follows:

1 $$ I_{FD} \left( q \right) = R_{FD} \left( {\theta_{0} + \alpha \left( q \right)} \right) \cdot I\left( q \right) , $$

2 $$ I_{D} \left( q \right) = R_{D} \left( {\theta_{0} + \alpha \left( q \right)} \right) \cdot I\left( q \right) $$

where ${\theta }_{0}$ is the angle of incidence of the X-ray beam into the LAA when the sample is removed and $I\left(q\right)$ is the X-ray intensity including X-ray absorption in the sample. Although it is possible to separate the absorption components from ${I}_{FD}\left(q\right)$ and ${I}_{D}\left(q\right)$ by multiple measurements, for simpler derivation, we assume that $I\left(q\right)={I}_{0}$ under the assumption that the sample behaves as a pure phase object. $\alpha \left(q\right)$ is the angular deviation after the beam has propagated through the sample and represents the first derivative of the line integral of *δ* on the subject:

3 $$ \alpha \left( q \right) = \int\limits_{{ - \infty }}^{\infty } {\frac{{\partial \delta \left( {p,q} \right)}}{{\partial q}}dp}  $$

In conventional XDFI, the distance between the LAA and the detector is sufficiently large so that the FD and D images do not overlap, and only the FD signal is measured when little absorption contrast is observed, such as in biological soft tissue. Given that the refraction angle $\alpha \left(q\right)$ is very small, the observed signal can be described as follows:

4 $$  \begin{aligned}   I_{{FD}} \left( q \right) &  \cong I_{0} \left( {R_{{FD}} \left( {\theta _{0} } \right) + \frac{{dR_{{FD}} }}{{d\theta }}\alpha \left( q \right)} \right) \\    \quad  &  \equiv C\int\limits_{{ - \infty }}^{\infty } {\frac{{\partial \delta \left( {p,q} \right)}}{{\partial q}}dp + D}  \\  \end{aligned}  $$

where $C={I}_{0}\frac{d{R}_{FD}}{d\theta }$ and $D={I}_{0}{R}_{FD}\left({\theta }_{0}\right)$ are constants and can be estimated from the rocking curve. Thus, XDFI can measure the first derivative of the line integral of *δ* as in Eq. (4). SWIDeX acquires an image superimposing ${I}_{FD}\left(q\right)$ and ${I}_{D}\left(q\right)$ by placing the scintillator immediately after the LAA. Defining $f\left(\theta \right)={I}_{0} {R}_{FD}\left(\theta \right)$ and $g\left(\theta \right)={I}_{0} {R}_{D}\left(\theta \right)$ in $\theta \in {\Theta }_{D}$, where ${\Theta }_{D}$ is the region used for measurement where the rocking curve is monotonic. Equations (1) and (2) are represented as ${I}_{FD}\left(q\right)=f\left({\theta }_{0}+\alpha \left(q\right)\right)$, ${I}_{D}\left(q\right)=g\left({\theta }_{0}+\alpha \left(q\right)\right)$, and $f\left(\theta \right)+g\left(\theta \right)={I}_{0}$ from the energy conservation law. If ${I}_{FD}\left(q\right)$ and ${I}_{D}\left(q\right)$ completely overlap, the refraction contrast disappears, but here we consider the situation where the LAA and the scintillator are separated by a small distance $\Delta p$. Due to the $\Delta p$ gap, ${I}_{D}\left(q\right)$ shifts in the q direction by $\Delta q=\Delta p\text{tan}2{\theta }_{B}$, so the X-ray intensity observed on the scintillator is

5 $$ I_{SX} \left( q \right) = I_{FD} \left( q \right) + I_{D} \left( {q - \Delta q} \right) = f\left( {\theta_{0} + \alpha \left( q \right)} \right) + g\left( {\theta_{0} + \alpha \left( {q - \Delta q} \right)} \right) = f\left( {\theta_{0} + \alpha \left( q \right)} \right) - f\left( {\theta_{0} + \alpha \left( {q - \Delta q} \right)} \right) + I_{0} $$

Substituting $f\left( {\theta_{0} + \alpha \left( {q - \Delta q} \right)} \right) \cong f\left( {\theta_{0} + \alpha \left( q \right)} \right) - \frac{df}{{d\theta }}\frac{d\alpha }{{dq}}\Delta q$ to Eq. (5),

6 $$ I_{SX} \left( q \right) \cong \frac{df}{{d\theta }}\frac{d\alpha }{{dq}}\Delta q + I_{0} = C^{\prime } \frac{d\alpha }{{dq}} + I_{0} $$

where $C^{\prime } = \Delta q\frac{df}{{d\theta }}\left( {\theta_{0} + \alpha \left( q \right)} \right)$, which can be regarded as a constant, i.e., $\frac{df}{{d\theta }}\left( {\theta_{0} + \alpha \left( q \right)} \right) \approx \frac{df}{{d\theta }}\left( {\theta_{0} } \right)$. This expression is a first-order approximation; a higher-order approximation may be used to represent the output signal with higher precision.

Thus, Eq. (6) is given as

7 $$  I_{{SX}} \left( q \right) = C^{\prime } \int\limits_{{ - \infty }}^{\infty } {\frac{{\partial ^{2} \delta \left( {p,q} \right)}}{{\partial q^{2} }}dp + I_{0} ,}   $$

This means that the contrast of the projection formed on the scintillator represents the second derivative of the line integral of *δ*. It is also generally known that the Radon transform of a Laplacian image is a second derivative projection:

8 $$  \mathop \int \limits_{ - \infty }^{\infty } \Delta \delta \left( {p\cos \varphi - q\sin \varphi ,p\sin \varphi + q\cos \varphi } \right)dp = \mathop \int \limits_{ - \infty }^{\infty } \left( {\frac{{\partial^{2} }}{{\partial x^{2} }} + \frac{{\partial^{2} }}{{\partial y^{2} }}} \right)\delta dp = \mathop \int \limits_{ - \infty }^{\infty } \left( {\frac{{\partial^{2} }}{{\partial p^{2} }} + \frac{{\partial^{2} }}{{\partial q^{2} }}} \right)\delta dp = \mathop \int \limits_{ - \infty }^{\infty } \frac{{\partial^{2} \delta }}{{\partial q^{2} }}dp + \left[ {\frac{\partial \delta }{{\partial p}}} \right]_{ - \infty }^{\infty } = \mathop \int \limits_{ - \infty }^{\infty } \frac{{\partial^{2} \delta }}{{\partial q^{2} }}dp,  $$

After obtaining the projection of Eq. (7) from various directions of the subject using SWIDeX, the Laplacian image with high sparsity is obtained by reconstruction of these projection views.

In conventional XDFI, the projection representing the first derivative is acquired as shown in Eq. (4), so the Laplacian image reconstruction requires prior derivative processing in the *q* direction. This process is carried out by software differencing, which leads to noise amplification and spatial resolution degradation. With SWIDeX, Eq. (7) can be obtained directly, eliminating the need for extra preprocessing.

### Limited-view reconstruction algorithm using second derivative projection of SWIDeX

The projection reduction by ART + TV method is effective in reconstructing CT images with high sparsity, and it is expected to have a high projection reduction effect when applied to the second-order differential projection of SWIDeX. We describe a method for reconstructing a Laplacian image $\Delta \delta (x,y)$ of *δ* using the ART + TV method. First, a digital image $D(m,n)$ $(1\le m,n\le K)$ of $\Delta \delta (x,y)$ is introduced. Also, *N*-dimensional vector representing $D(m,n)$ is defined as $\text{D}=({d}_{1},{d}_{2},\cdots ,{d}_{N})$, where $N=K\times K$ and ${d}_{mK+n}=D(m, n)$. Next, Eq. (2) is digitized as follows:

9 $$ \begin{array}{*{20}c} {\mathop \sum \limits_{j = 1}^{N} w_{ij} d_{j} = h_{i} } & {i = 1,2, \cdots M} \\ \end{array} $$

where ${h}_{i}$ corresponds to the right-hand side of Eq. (8). *M* is the total number of rays, and the weight matrix ${w}_{ij}$ is equal to the line segment where the *i*-th ray crosses the *j*-th pixel and represents the contribution of the *i*-th line integral to the *j*-th pixel. Equation (9) can be expressed as ${\mathbf{WD}} = {\mathbf{h}}$ where **W** is an $M\times N$ matrix with ${w}_{ij}$ as the (*i*, *j*)th element and h is an *M*-dimensional vector with ${h}_{i}$ as the *i*-th component. We formulate the reconstruction for **D** with TV regularization as follows:

10 $$ \mathop {\min }\limits_{D} ||{\mathbf{WD}} - {\mathbf{h}}||^{2} \, +\, \lambda TV\left( {\mathbf{D}} \right) $$

11 $$ TV\left( {\mathbf{D}} \right) = \sum\limits_{{m,n}} {\sqrt {\left| {d\left( {m + 1,n} \right) - d\left( {m,n} \right)} \right|^{2}  + \left| {d\left( {m,n + 1} \right) - d\left( {m,n} \right)} \right|^{2} } }   $$

where $\lambda$ controls the relative weights of the data fidelity and regularization terms.

To obtain the reconstructed image, the method proposed in [25] is applied. The algorithm obtains a solution from the minimization problem (10) by alternately repeating the ART step and the TV minimization step. In the ART step, the standard ART algorithm reconstructs the image by iteration. The reconstructed image output by the ART step is then supplied to the TV step as the initial value. In the TV step, the objective function in Eq. (11) is minimized using the standard steepest descent method with derivatives of the TV terms and D is the solution of $\Delta \delta$ when Eq. (10) falls below a certain threshold.

Finally, $\delta \left(x,y\right)$ is obtained by solving numerically the Poisson equation $\left({\partial }^{2}/\partial {x}^{2}+{\partial }^{2}/\partial {y}^{2}\right)\delta \left(x,y\right)=\widetilde{\Delta \delta }\left(x,y\right)$, where $\widetilde{\Delta \delta }$ is a Laplacian image, under the Dirichlet boundary condition that $\delta \left(x,y\right)=0$ for $\left(x,y\right)\in \partial\Omega$. To solve the Poisson equation, we use the successive over-relaxation (SOR) method [26]. The three-dimensional δ distribution of an object can be obtained by stacking two-dimensional CT reconstruction images.

## Experiments and results

### Simulation

**Fig. 2:**
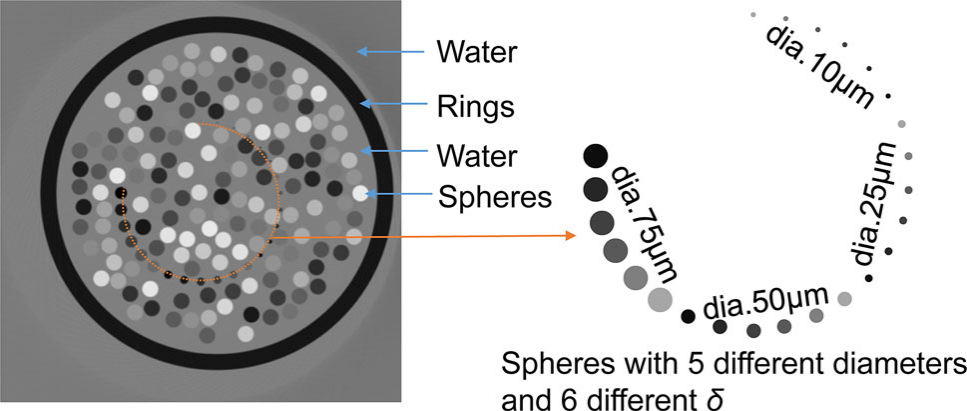
A schematic view of the numerical phantom in the simulation experiment

First, the projection reduction effect in an ideal condition is verified using a SWIDeX-CT imaging simulator based on geometric optics that reflects the physical parameters in real experiments developed by us. In this simulator, the x-ray beam propagates on the numerical phantom in parallel and is refracted according to Snell's law at the refractive index boundary on the numerical phantom. The refraction angle of each beam is converted to FD and D intensities using the rocking curve of LAA and stored as a superimposed projection as in Eq. (5). To evaluate the effect of the number of projections on image quality, measurement noise and absorption by the sample are not modeled. The numerical phantom consists of rings, water, randomly placed spheres, and circularly placed spheres as shown in Fig. 2. The ring has a diameter of 2.2 mm, a thickness of 0.2 mm, and a δ value of 6.0 × 10^−7^, which is close to that observed in tubular biological soft tissue. It is positioned 0.05 mm to the right and 0.1 mm parallel to the upper side from the center of the reconstruction. Water is placed outside and inside of it, and the δ of water is set to 5.75 × 10^−7^. Inside the ring, spheres with diameters of 0.1 mm and *δ* ranging from 5.5 × 10^−7^ to 6.0 × 10^−7^ are placed in random positions. Furthermore, to evaluate spatial resolution and contrast, spheres consisting of six different *δ* (0.05 × 10^−7^ steps from 5.80 × 10^−7^ to 6.05 × 10^−7^) and five different diameters (see Fig. 2 right) are placed in a circle with a radius of 0.5 mm from the reconstruction center. These refractive index values assume an incident X-ray energy of 20.0 keV. The number of bins in one projection is 500, the matrix size of the reconstructed image is 500 × 500 pixels, and the pixel dimensions are 5 × 5 µm^2^. Second derivative projections are generated at constant angular steps over a span of 180 degrees.

To investigate the effectiveness of ART + TV on second derivative projections, we compared it to FBP and ART without TV regularization; the number of iterations for both ART and ART + TV was 100 with sufficient convergence of the reconstructed images.

**Fig. 3:**
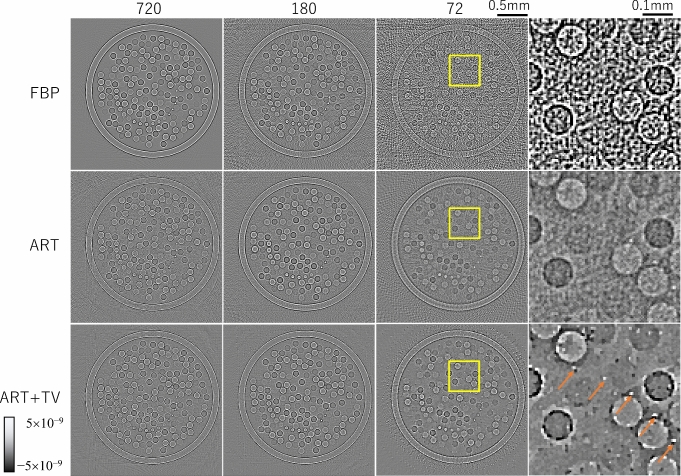
Laplacian *δ* images reconstructed using the FBP, ART, and ART + TV algorithms for different numbers of projections. The right column shows a magnified image in the square region of each reconstructed image using 72 views

**Fig. 4:**
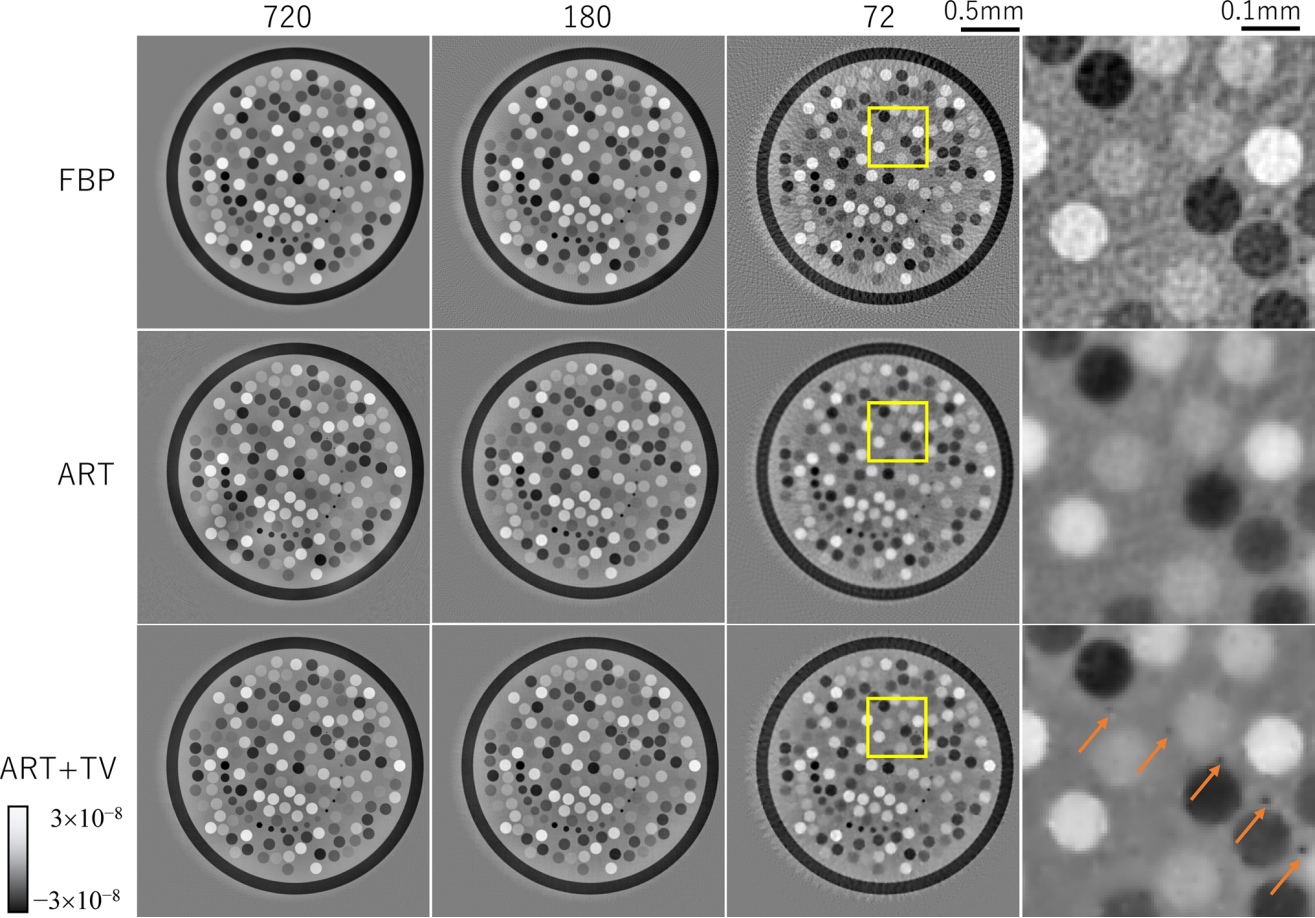
*δ* images obtained by applying the SOR method to each Laplacian δ image in Fig. 3. The right column shows a magnified image in the square region of each reconstructed image using 72 views. The orange arrows show small spheres of 10 µm diameter placed in the numerical phantom

Figure 3 shows reconstructed images in 720, 180, and 72 projection views using FBP, ART, and ART + TV. According to the sampling theorem, reconstruction using 500 detector bins requires 720 views. A subjective evaluation of image quality indicates that FBP provided satisfactory reconstruction at 720 views, but significant stripe artifacts appeared in the image at 180 views, which is a quarter of the number of views. ART tended to emphasize noise slightly more than FBP. When the number of projection views was reduced, the effect of stripe artifacts was also apparent, but appeared milder than with FBP. ART + TV offered a slightly sharper image than FBP at 720 views and produced acceptable image quality even when the number of projection views was reduced. The right column of Fig. 3 shows a magnified image in the square region of each reconstructed image using 72 views. ART + TV was able to delineate spheres with a diameter of 10 µm and a refractive index difference of 0.1 ~ 0.3 × 10^−7^ with high contrast, as indicated by the arrows. Figure 4 is CT images of *δ* obtained by applying the SOR method to each of the reconstructed images in Fig. 3. Each δ image has fewer artifacts than each corresponding Laplacian image because the small variations in the Laplacian image are integrated by the process of the SOR method. Among them, ART + TV offers the best image quality.

**Fig. 5:**
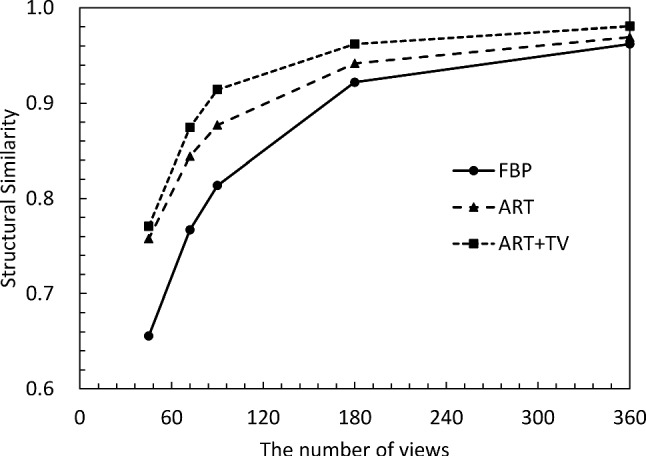
Dependence of structural similarity on the number of projections

**Fig. 6:**
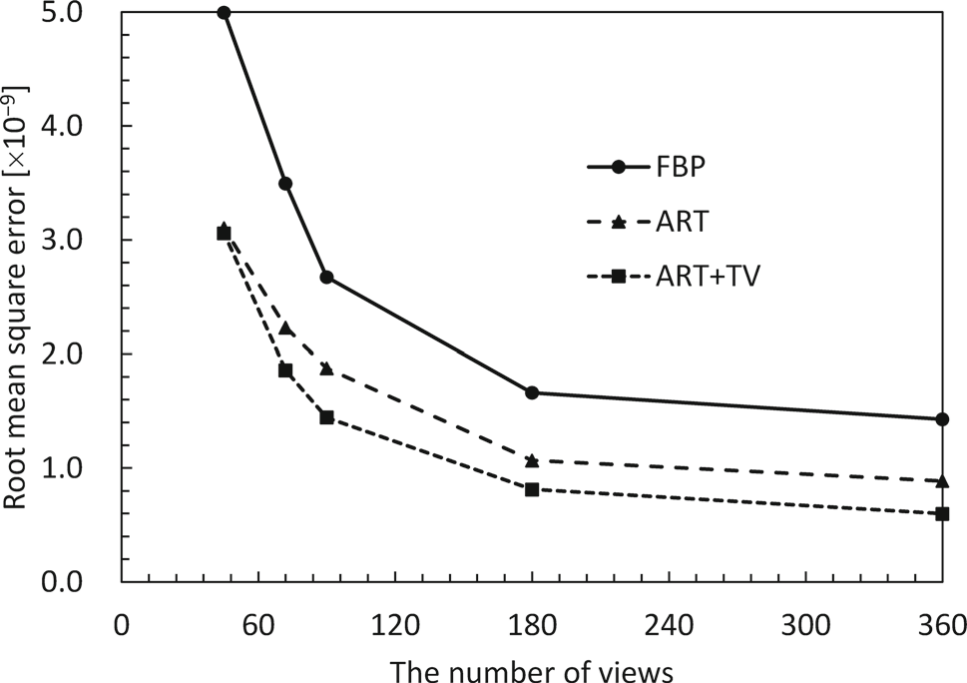
Dependence of root-mean-square error on the number of projections

Finally, the quality of the CT reconstructed images was quantitatively evaluated. Figures 5 and 6 show the structural similarity (SSIM) [27] and the root-mean-square error (RMSE) of the *δ* images versus the number of projection views, respectively. The SSIM and RMSE of the FBP start to decrease rapidly around 180 views, because it violates the sampling theorem. On the other hand, ART and ART + TV deteriorate their respective values more slowly than FBP. Also, ART + TV maintains SSIM values above 0.9 even with only 90 views; the behavior of SSIM and RMSE is consistent with the subjective assessments in Figs. 3 and 4. From these results, we can conclude that the quality of ART + TV is superior to FBP and ART.

### Experiment

**Fig. 7:**
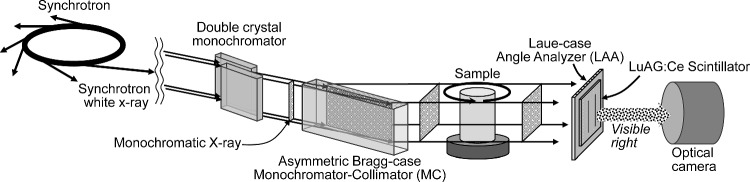
Schematic diagram of the SWIDeX-CT imaging system

The effectiveness of the proposed method was investigated using actual projection data obtained from a CT measurement system based on SWIDeX. Figure 7 is an overview diagram of the imaging system, which consists of an asymmetric cut Bragg-case monochromator collimator (MC), a sample rotation stage, an asymmetric cut LAA, an X-ray scintillator, and an optical camera, and was installed at the BL14B beamline of the High Energy Accelerator Research Organization Photon Factory (KEK-PF) in Tsukuba, Ibaraki, Japan. Synchrotron radiation X-rays are first diffracted at the MC, which increases the horizontal beam size and reduces the divergence angle. The beam is then refracted according to the complex refractive index described above as it propagates through the sample, resulting in an angular deviation of approximately 10^−7~8^ radians. The angular deviation of the beam is modulated by the X-ray diffraction of the LAA to the X-ray intensity, forming a differential refraction-contrast image at the back of the LAA, i.e., the second derivative of the line integral of *δ*. This image is converted to visible light by an X-ray scintillator and acquired by an optical camera. Projections required for CT reconstruction are obtained by repeatedly rotating and taking images of the subject. Synchrotron radiation X-rays are generated from the 2.5 GeV storage ring at KEK-PF and produced by a 5.0 T superconducting wiggler operating in a horizontal magnetic field. Since the polarization of the emitted beam is perpendicular, the diffraction plane of the Si crystal optics and the rotation axis of the subject are arranged to be perpendicular to the ground. The X-ray source size is 1060 µm horizontally and 90 µm vertically [28], and the X-ray energy monochromatized by the double-crystal monochromator is 19.8 keV. The intensity of X-rays in the FD direction for Bragg angle incidence depends on X-ray energy and the thickness of the LAA, and the closer the intensity is to 0, the higher the refractive contrast obtained. However, in this experiment, there was a discrepancy between the calculated and actual thickness, which required a slight adjustment of the X-ray energy from 20 keV to compensate for the discrepancy. Both MC and LAA use Si (111) diffraction planes with a Bragg angle of 5.73 degrees. The asymmetry angle of the MC is 5.4 degrees, and the asymmetry factor $b=\text{sin}(5.73-5.4)/\text{sin}(5.73+5.4)\cong 0.03$. The field of view and flux of the beam according to MC are approximately 23 × 21 mm^2^ and 10^8^ photons/mm^2^/s, respectively. The asymmetry angle of the LAA is approximately 5 degrees and the crystal thickness is 166 μm, allowing the camera to be aligned nearly parallel to the beam path. By rotating the LAA a small angle of − 0.5 arcsec from the Bragg angle, refraction contrast is generated by the steep slope of the rocking curve of LAA. The scintillator, which converts X-rays into visible light, is placed against the LAA, and there is almost no gap between the LAA and the scintillator. The scintillator is a LuAG:Ce (Lu_3_Al_5_O_12_:Ce) crystal with a thickness of 100 μm. The distance between the LAA and the scintillator is approximately 8 µm. The camera used is a Hamamatsu Photonics K.K. model C14120-20P with a pixel size of 5.5 µm, a sensor size of 25.344 × 14.256 mm^2^, a pixel matrix of 4608 × 2592 , and a dynamic range of 16 bits.

The specimen to be imaged by SWIDeX is a surgically resected specimen from a human patient with intraductal papillary mucinous neoplasia (IPMN). This specimen has been formalin-fixed, ethanol-preserved, and pathologically diagnosed. A few hours before CT imaging, the ethanol in the specimen is replaced with distilled water and placed in a cylindrical plastic container filled with agarose gel with an outer diameter of 22 mm and an inner diameter of 20 mm. The number of projection views is 1250 and the rotation angle step between projections is 0.144 degrees; the exposure time per projection is 4 s and the total CT imaging time is approximately 1.5 h. Since there are 4608 detector elements in the horizontal direction, approximately 7000 views are required according to the sampling theorem. The number of 1250 views in this experiment is much less than the theoretical requirement, but it is the maximum number that could be obtained within the limited beamtime.

**Fig. 8:**
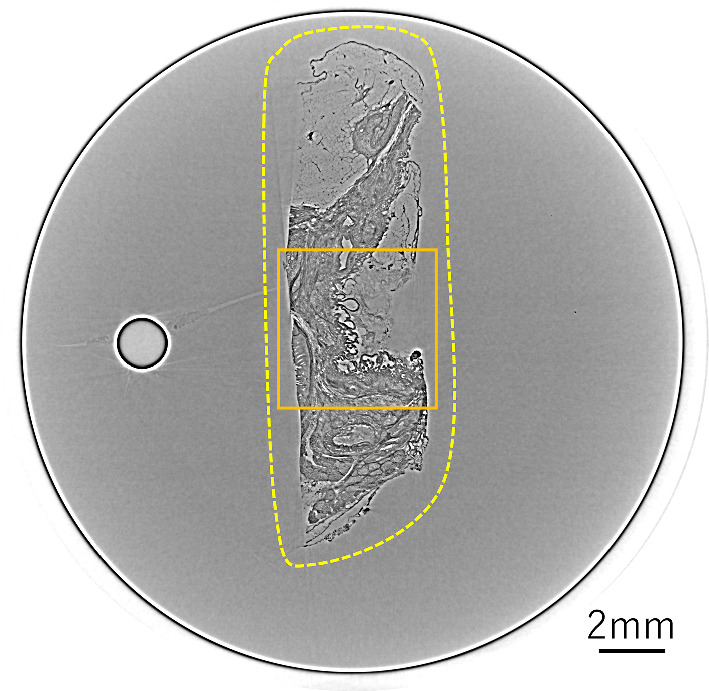
The reconstructed image of the *δ* distribution obtained by applying FBP to the projection of 1250 views. The area enclosed by the yellow dotted line represents a cross section of the IPMN tissue inserted into the cylindrical container. The orange square is the area used to calculate SSIM and RMSE

**Fig. 9:**
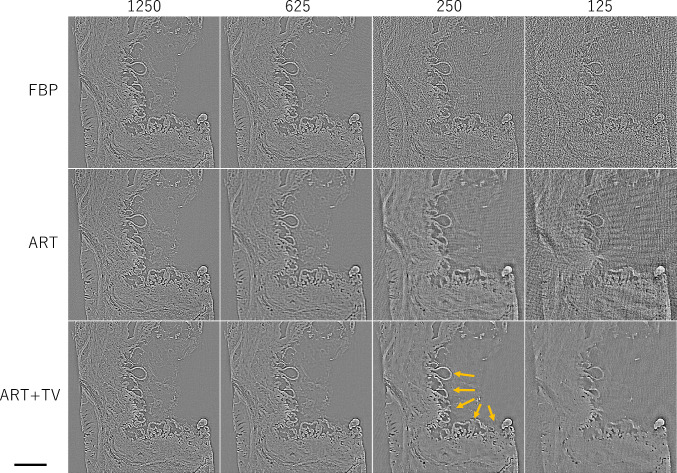
Laplacian *δ* images reconstructed using the FBP, ART, and ART + TV algorithms for different numbers of projections. The vesicular structure indicated by the orange arrow is papillary-like tumor tissue caused by IPMN. Scale bar is 100 µm

**Fig. 10:**
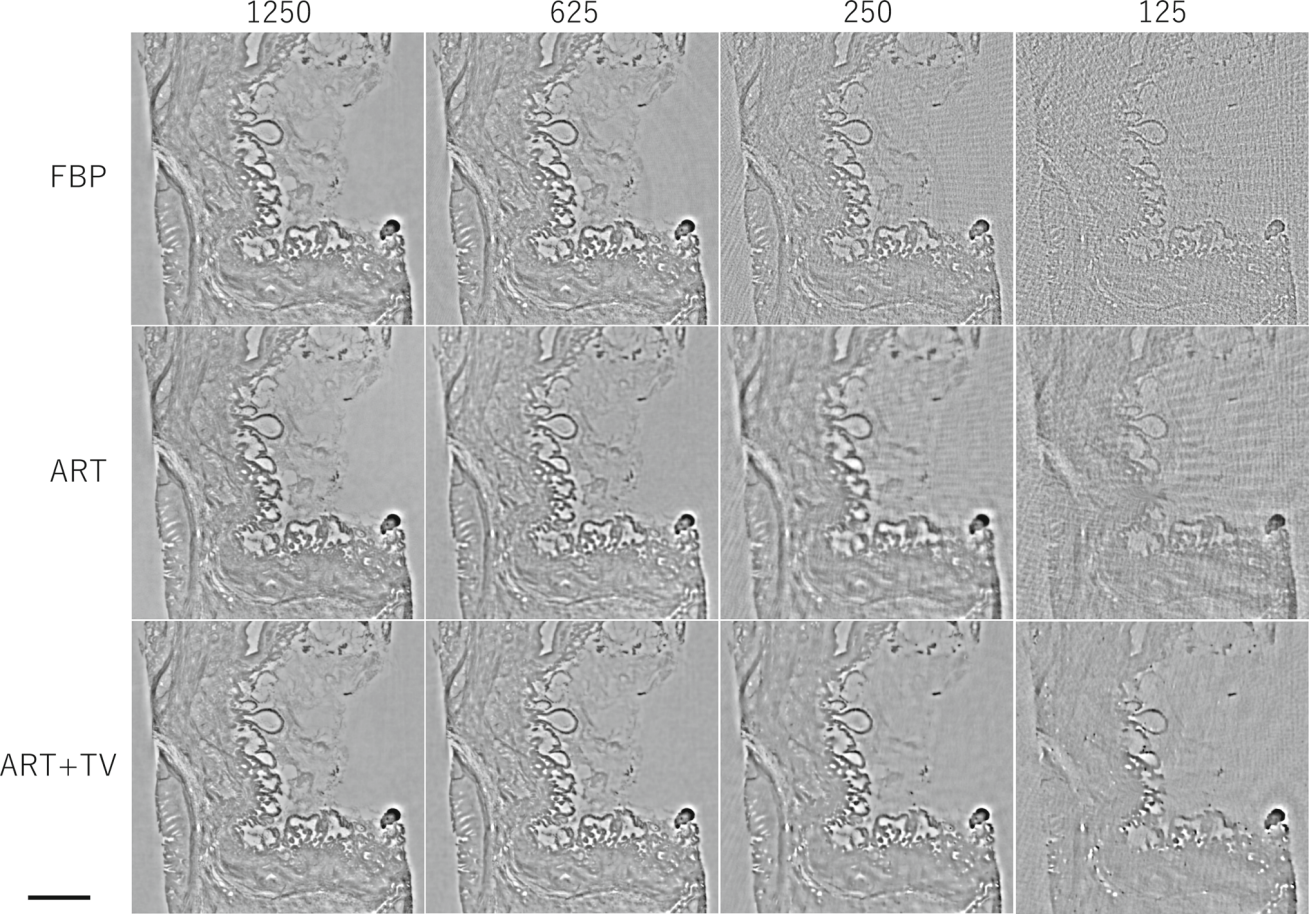
*δ* images obtained by applying the SOR method to each Laplacian δ image in Fig. 8. Scale bar is 100 µm

**Fig. 11:**
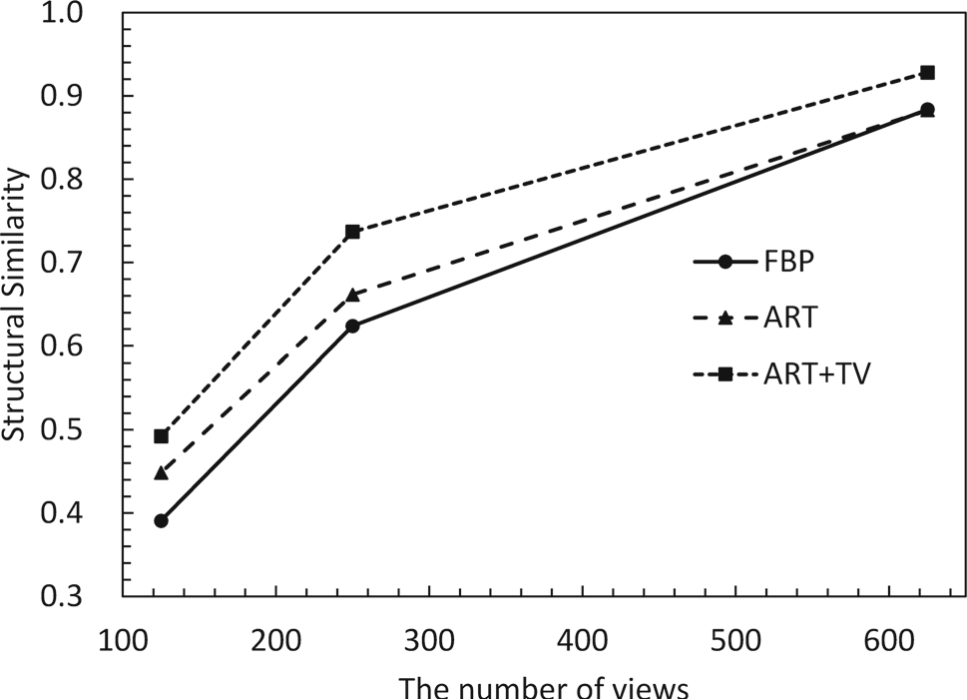
Dependence of structural similarity on the number of projections

**Fig. 12:**
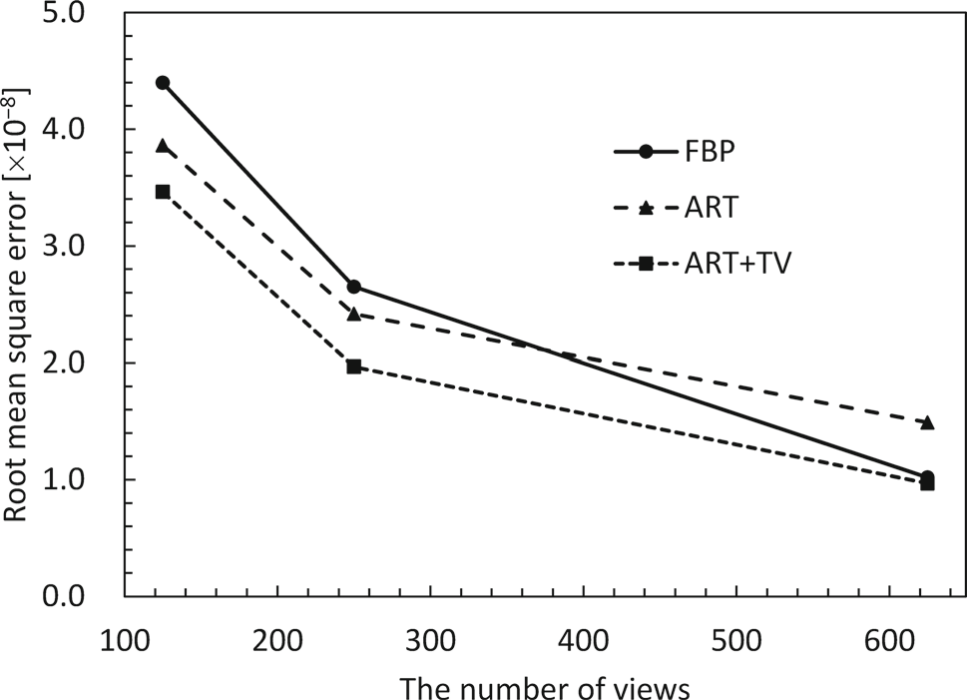
Dependence of root-mean-square error on the number of projections

Figure 8 depicts the reconstructed image of the δ distribution obtained by applying FBP to the projection of 1250 views. The area enclosed by the yellow dotted line represents a cross section of the IPMN tissue inserted into the cylindrical container. Figure 9 compares Laplacian δ images reconstructed using varying numbers of projections and different reconstruction methods, and the orange square area in Fig. 8 is enlarged to facilitate a more detailed comparison of the differences in image quality. A subjective observation indicates that ART + TV provides good image quality even at 250 views, with clear delineation of the bubble-like papillary structures produced by the IPMN, as indicated by the orange arrow. However, in the 125 views, the streak-like artifacts is reduced, but the tissue structure is obscured. Figure 10 shows the *δ* images obtained by applying the SOR method to each of the reconstructed images in Fig. 9. From these images, ART + TV provides the best image quality as in Fig. 9. Figures 11 and 12 show the dependence of SSIM and RMSE on the number of projections, respectively. The SSIM and RMSE values are calculated from the entirety of the reconstructed image in Fig. 10, that is, within the orange square area in Fig. 8. For all images, the indices deteriorate sharply from 250 views, but the indices for ART + TV are better than those for FBP and ART. These results show that ART + TV is superior to FBP and ART in image quality of CT reconstructed from a limited number of views.

## Discussion

Simulation and actual experimental results of X-ray CT imaging showed the effectiveness of the projection reduction of the ART + TV method based on the second derivative projections obtained by SWIDeX. In simulations, image quality was maintained even when the number of projections was reduced to about 1/10 of the theoretically required number, and in actual experiments, tumor structures could be delineated even when the number of projections was reduced to about 1/30. This is due to the high sparsity of the second derivative projections obtained by SWIDeX, as described in the Methods section. Conventional X-ray CT, which uses the line integral of the physical distribution as a projection, often has complex textures that are not sparse on the object, and ART + TV can lead to excessive flattening. In SWIDeX, the FD and D images representing the first derivative diffracted behind the LAA are spatially superimposed to form a second derivative projection, so that the object information on the projection is concentrated at the boundaries of the refractive index difference, resulting in sparser values than the original *δ* distribution. This achieves a high projection reduction effect. On the other hand, subjects with microstructures that have a large variation in refractive index difference may have less sparsity in SWIDeX and the projection reduction effect may not be as great as expected. The numerical phantom used in the simulation was composed of many microspheres to simulate such a situation. The reason why the actual experimental results are more effective in reducing the number of projections than the simulation is thought to be because the flat area in the reconstruction space is larger in the experimental results. Thus, in actual experiments, the number of projections could be adjusted by making some assumptions about the complexity of the refractive index within the subject.

## Conclusion

In this study, we demonstrated the projection reduction effect of the ART + TV method based on SWIDeX from a simulation and an actual X-ray CT imaging experiment. This technique will be useful in the future for CT reconstruction from large differential projection data with wide field of view and high spatial resolution.

## Data Availability

The datasets generated and analyzed during the current study are available from the corresponding author on reasonable request.
